# Stabbing on Six Mile: A Case Report of Tension Pneumopericardium Following Penetrating Trauma

**DOI:** 10.7759/cureus.27172

**Published:** 2022-07-23

**Authors:** Nathaniel Reed, Zachary J Brennan, Jason Kurle

**Affiliations:** 1 Surgery, Detroit Medical Center, Detroit, USA; 2 Surgery, Michigan State University College of Osteopathic Medicine, East Lansing, USA

**Keywords:** thoracic trauma, penetrating trauma, tamponade physiology, tamponade pneumopericardium, tension pneumopericardium

## Abstract

This report details a rare case of cardiac tamponade due to traumatic pneumopericardium in a 27-year-old male. The patient presented to the emergency department in May 2021 as a Level 1 trauma activation due to multiple stab wounds, including one in the epigastric region, with tachycardia and hypotension. He was found to have significant pneumopericardium, resulting in tamponade physiology. The patient was emergently transferred to the operating room for a left anterolateral thoracotomy with pericardiotomy. He recovered well postoperatively without complications and was discharged home on postoperative day four.

## Introduction

Cardiac tamponade is caused by an abnormal increase in fluid accumulation in the pericardial sac, which, by raising intracardiac pressures, impedes normal cardiac filling and reduces cardiac output; cardiogenic shock and death can occur due to reduced inflow gradients [1]. Cardiac tamponade can be caused by pneumopericardium; air tamponade has been reported with a high mortality rate of 56% [2]. It classically presents with Beck’s triad, which consists of hypotension, muffled heart sounds, jugular venous distension, as well as pulsus paradoxus [3]. Transthoracic echocardiogram (TTE) is the gold standard for diagnosis, and management includes pericardiocentesis or pericardiostomy, depending on the clinical picture [3].

According to most sources, pneumopericardium in blunt trauma is caused by alveoli rupture due to a sudden rise in intrathoracic pressure, which leads to an air leak to the pericardium via the pleural cavity, though another mechanism would consist of direct apposition of tracheobronchial and pericardial tears. In addition, the spread of air can occur via tracking along perivascular planes of the pulmonary vessels into the mediastinum or pericardium, termed the “Macklin Effect” [4]. Non-tension pneumopericardium is when pericardial pressures are higher than the normal 50-100mmHg but less than 145mmHg; tension pneumopericardium is when pressures exceed 145mmHg [5]. Tension pneumopericardium, while rare, is associated with trauma and positive pressure ventilation, and may lead to cardiac tamponade [6]. Tension pneumopericardium is reported in one study to occur in approximately 37% of pneumopericardium patients. The development of these conditions is particularly concerning as the mortality rate of pneumopericardium or tension pneumopericardium is nearly 57% according to the same study [2].

## Case presentation

**Figure 1:**
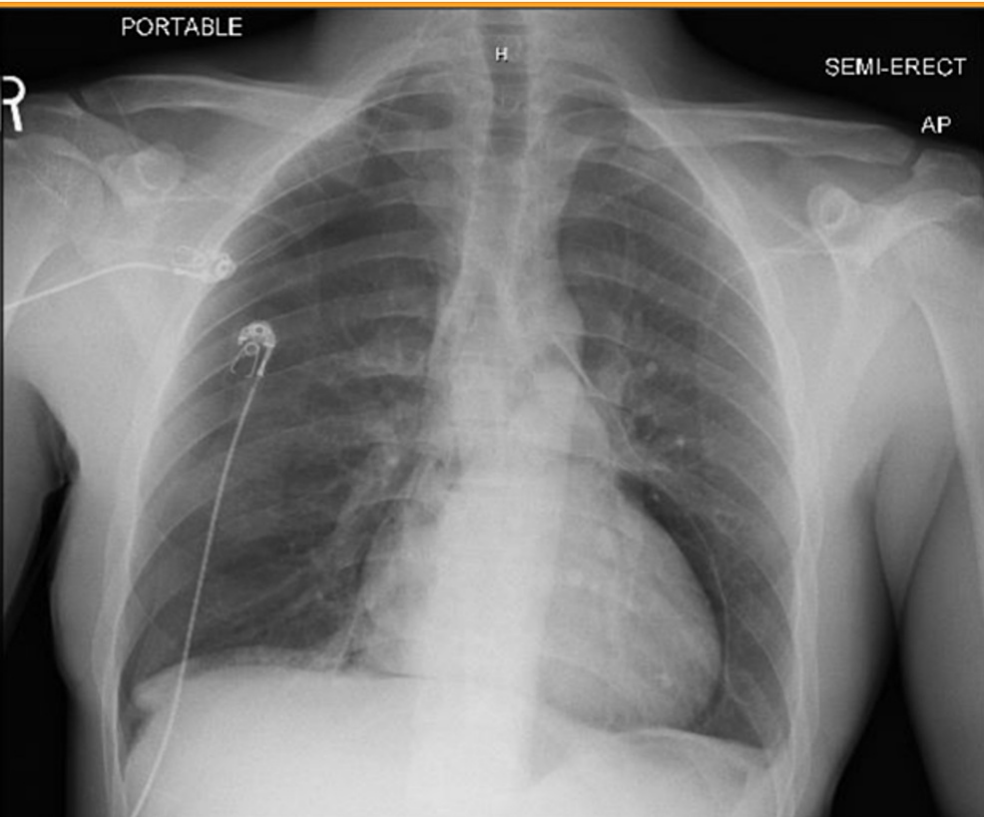
Initial chest x-ray on presentation to the emergency department

The patient was a 27-year-old male with no significant past medical history who presented to the emergency department as a Level 1 trauma activation due to multiple stab wounds to bilateral forearms and epigastrium (Figure 1). The patient was displaying signs of hemodynamic instability with tachycardia > 130 beats per minute and hypotension with systolic blood pressures around 90s mmHg. The patient was notably disoriented and in obvious distress. He was tachypneic with bilateral breath sounds. Oxygen saturation was 92% on room air and improved with the placement of a nonrebreather mask. Massive transfusion protocol was initiated due to concern for hemorrhagic shock as a cause of the patient’s hypotension and tachycardia. It was stopped following the diagnosis of tension pneumopericardium. The patient was transfused one unit of packed RBC, one unit of fresh frozen plasma, and one dose of tranexamic acid (TXA) in total. The pericardial window of the focused assessment with sonography in trauma (FAST) exam was non-diagnostic due to the presence of air obscuring cardiac views and the remainder of the exam was normal with no intra-abdominal fluid identified. Chest x-ray then revealed pneumopericardium and a small right pneumothorax (Figure 1).

Given the absence of hemothorax and the small size of the right pneumothorax without shifting of mediastinal structures, pneumopericardium resulting in tamponade physiology was deemed the most probable cause of the patient’s hemodynamic instability. The decision was made to defer treatment of right pneumothorax and the patient was taken directly to the operating room for pericardiotomy, as taking time for right chest thoracostomy in the trauma bay would have delayed the treatment of cardiac tamponade and resulted in further deterioration. Despite imaging findings of right-sided processes, a right thoracotomy approach would have limited exposure to the heart, which is necessary given the unknown extent of the injury. While a median sternotomy would provide excellent access to mediastinal structures, access to the pleural cavities is limited with this approach. Thus, the author’s preferred approach was left anterolateral thoracotomy for access in traumatic cardiac injuries as this provides rapid access to the heart and pleural space with the option for right-sided video-assisted thoracoscopic surgery (VATS) or conversion to clamshell thoracotomy if access to the right pleural space is required. 

Left anterolateral thoracotomy revealed dense, chronic adhesions throughout the left hemithorax, as well as pneumopericardium. There was no obvious hemopericardium. The chronic pleural adhesions obscured complete access to the pericardium initially. A small incision was made in the pericardium at an accessible location to relieve tension. Upon pericardiotomy and release of pericardial tension, the patient’s hemodynamic instability immediately improved, confirming tension pneumopericardium as the primary cause. Left total lung decortication was performed to evaluate for injury and aid in exposure. Completion pericardiotomy and mediastinal exploration revealed no injury to the heart or great vessels. Lab work obtained in the trauma bay before proceeding to the operating room resulted intraoperatively and revealed a hemoglobin level of 13.1 with no other abnormalities, further transfusions were not required. There was a 1 cm right-sided pericardial laceration, which was repaired with absorbable suture. The epigastric wound was also explored and a small right diaphragmatic injury was noted. A portion of the right lower lobe was herniated through the wound and was reduced. Due to the lung herniation and right-sided pericardial injury, a right-sided VATS was performed for local exploration of the affected hemithorax.

**Figure 2:**
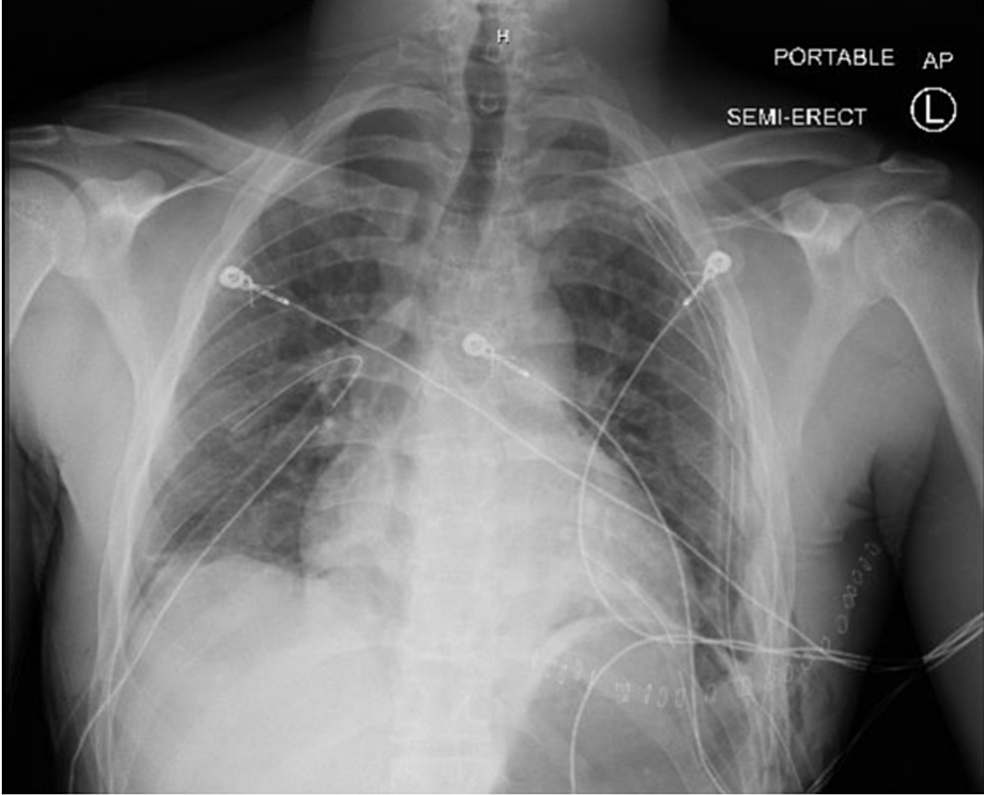
Postoperative chest x-ray demonstrating resolution of the pneumopericardium

When performing diagnostic VATS for penetrating injury, it is the author’s preference to initially utilize existing wounds for access with conversion to more standardized trochar placement if further intervention is deemed necessary. A 12 mm trocar was placed through the epigastric wound and into the right hemithorax. A second trocar was placed in the right midaxillary line and the camera was inserted. A small hemothorax was evacuated, the pericardial injury was noted, and no other injuries were identified. A right chest tube was left in place and the pericardial laceration was closed. The epigastric wound was closed in layers to prevent future lung herniation. The diaphragm was also repaired. There was no air leak noted following repair. Pericardiotomy was then closed with running absorbable suture. After thoracotomy closure, a diagnostic laparoscopy revealed no intra-abdominal injuries. The left wrist wound was then addressed and the transected ulnar artery ligated after confirmation of an intact palmar arch. The patient was extubated and transferred to the surgical intensive care unit (SICU) in stable condition. Postoperative x-rays demonstrated resolution of the pneumopericardium and pneumomediastinum with a small apical right pneumothorax (Figure 2).

Resolution of the pneumothorax was noted on postoperative day two and the chest tubes were placed to water seal. Chest tubes were removed on postoperative day three without recurrence of pneumothorax or pneumopericardium. The patient recovered well without complications and was discharged home on postoperative day four. The patient followed up in the trauma surgery clinic and was recovering well without complications from his thoracotomy.

## Discussion

Pneumopericardium is a rare finding and can be seen in cases of both penetrating and blunt injuries. Even more rarely does it result in tamponade physiology, placing the patient in a critical condition. The prompt identification of this unusual pathology is essential to a successful outcome for the patient, especially considering the high mortality rate associated with these injuries.

This case report demonstrates how our institution was able to quickly diagnose and address this pathology and provides an opportunity to illustrate the benefits of our approach. Our institution cares for a higher than average volume of penetrating trauma and therefore maintains an environment of high suspicion for unusual thoracic trauma pathology. As a result, the expedient performance of a chest radiograph, recognition of hemodynamic instability, and time from door to the operating room is exceptionally fast in our trauma bays and were vital to this patient’s survival. Right chest thoracostomy in the trauma bay is unlikely to result in decompression of the pericardium and relief of tamponade in this case. Although the operation did reveal the etiology of the pneumopericardium to be a laceration to the right pericardium via the right hemithorax, there was no shifting of mediastinal structures to indicate tension pneumothorax as a cause for his instability. Additionally, if the pericardial laceration was not obstructed by clots or another structure, the pneumopericardium would preferentially decompress into the right hemithorax before resulting in tamponade physiology and hemodynamic instability. Due to this, pneumopericardium should be considered as a separate entity from pneumothorax when considering treatment options in the hemodynamically unstable patient. Ensuring that advanced trauma life support (ATLS) protocols are followed efficiently and that an operating room is immediately available are essential to the care of critical thoracic trauma such as tension pneumopericardium.

Importantly, this case also illustrates several aspects of management that are not well discussed in the existing literature about traumatic pneumopericardium. One lesson from this report is the possibility for an unreliable FAST exam when diagnosing pneumopericardium in the trauma patient, thus highlighting the reliability of chest x-rays. Our institution recommends that the chest x-ray should not be delayed in cases of obvious thoracic trauma for other adjuncts, as it can often provide a more complete evaluation of intrathoracic pathology than a FAST exam. The FAST exam is largely focused around intra-abdominal pathologies. This makes it ideal for evaluation of patients with evidence of abdominal trauma. However, in cases of thoracic or thoracoabdominal trauma, the most immediately life-threatening trauma pathologies involve the thorax. Therefore, evaluation with imaging of the chest should take priority when there is high suspicion for serious injury.

Lastly, this report demonstrates the advantages of the left anterolateral thoracotomy for management of traumatic pneumopericardium. Most of the literature describes using alternative approaches to the management of tension pneumopericardium such as subxiphoid pericardial window and median sternotomy [6]. Although these have often been utilized successfully in tension pneumopericardium due to various causes such as ventilatory barotrauma, our institution would advocate for a left anterolateral thoracotomy in cases of penetrating trauma. The left anterolateral thoracotomy approach enables greater speed of entry into the thoracic cavity, decompression of the pericardium, and adequate visualization of vital structures. In addition, it can be easily converted into a clamshell thoracotomy for patients requiring exploration of the right hemithorax or greater exposure of the heart and great vessels. This is particularly important in trauma patients as the extent of their injuries is typically unknown prior to surgery. Approaches such as subxiphoid or median sternotomy are rarely performed outside of cardiothoracic surgery, and therefore present challenges to non-specialized surgeons who may be unfamiliar with the procedure or necessary instruments [7]. As a further note on the values of this case, we demonstrated a novel strategy of trocar placement using the wound channel for video-assisted evaluation of the right hemithorax without conversion to clamshell thoracotomy or requiring additional incisions. These considerations are based on the experiences at our institution and may provide insight to trauma surgeons or centers with lower rates of penetrating or thoracic trauma.

## Conclusions

Traumatic tension pneumopericardium is an exceedingly rare condition, and prompt surgical intervention is essential when tamponade physiology is recognized. This case illustrates the management and operative strategies utilized at our institution to manage this type of injury. It also illustrates the importance of differentiating related injuries in the trauma patient when creating an approach for management. In this case, a stab wound caused a right pneumothorax, which progressed to a tension pneumopericardium due to an associated pericardial injury from the same mechanism. Although these processes were closely associated, not recognizing these as separate pathophysiologic mechanisms would have likely resulted in delay in treatment of the more immediate life-threatening tamponade. We hope that this report provides an example to reinforce the importance of prompt diagnosis and clarify our recommendations for a surgical approach to the management of tension pneumopericardium.
